# Washing Hands according to the WHO Guideline since the COVID-19 Outbreak in the Context of Medical Undergraduates at a Tertiary Care Center: A Descriptive Cross-sectional Study

**DOI:** 10.31729/jnma.5259

**Published:** 2020-12-31

**Authors:** Anuska Khadka, Saurav Dani

**Affiliations:** 1 Lumbini Medical College and Teaching Hospital, Palpa, Nepal

**Keywords:** *COVID-19 prevention*, *hand hygiene*, *medical students*

## Abstract

**Introduction:**

Hand washing is an important preventive measure to avoid transmission of Coronavirus Disease of 2019. Medical students should be acquainted with the World Health Organization's hand-washing guidelines and should follow them to break the chain of spread of the virus. This study aims to find the acquaintance of medical undergraduates with the guidelines and to find out if they have started implementing these guidelines since the corona-virus outbreak.

**Methods:**

This is a descriptive cross-sectional study, conducted among MBBS, BSc and PCL nursing students in first year to internship of a tertiary care hospital from May 2020 to August 2020, and ethical clearance was received from the Institutional Review Committee (ref no: IRC-LMC 11- D/020) of Lumbini Medical College and Teaching Hospital. Data collection was done through online questionnaires. Data analysis of the obtained information was done in Microsoft-excel. Point estimate at 95% Confidence Interval was calculated along with frequency and proportion for binary data.

**Results:**

Of 462 respondents, 265 (57.4%) (52.9-61.9 at 95% Confidence Interval) respondents followed the World Health Organization hand-washing guidelines during every hand wash. Among them 172 (37.2%) participants had learned the guidelines through awareness programs. The majority of respondents belonged to 20-25 age groups, 275 (59.5%), and the majority were pursuing an MBBS degree, 360 (77.9%).

**Conclusions:**

We conclude that a notable number of medical undergraduates have been acquainted with standard hand-washing guidelines since the corona-virus outbreak, but some of them still do not follow the guidelines practically. Therefore, effective and impactful awareness programs need to be launched to improve hand hygiene practices.

## INTRODUCTION

Coronavirus disease of 2019 (COVID-19) is an infectious disease caused by severe acute respiratory syndrome coronavirus-2 (SARS-CoV-2),^1,2,3^ identified in December 2019 in Wuhan, China, and has spread globally, resulting in an ongoing pandemic.^4^ The World Health Organization (WHO) has published advice which includes maintaining a meter distance,^5^ wearing a mask, and following hand-washing guidelines entitled, "Hand Hygiene: Why, How and When".^6^ Despite following precautions, over 18 million cases of COVID-19 have been identified to date (4th August 2020).^7,8^

Even in Nepal the COVID-19 cases are still on rise, and medical personals are also infected.^9,10^ Therefore, the tendency of hand-washing according to WHO guidelines in medical personals should be known but the information regarding this topic is lacking in the **context** of Nepal.

This study aims to find the prevalence of medical undergraduates who are acquainted with WHO hand-washing guidelines and if they have started implementing these guidelines since the COVID-19 outbreak in a tertiary care center.

## METHODS

This descriptive cross-sectional study was conducted through an online survey and questionnaires were distributed through email and social networks when the whole country was under lockdown due to the COVID-19 outbreak. The ethical clearance was taken from Institutional Review Committee (IRC) (ref no: IRC- LMC 11-D/020) before starting the survey on the target population, and the study was completed over four months, from May 2020 to August 2020. The study population included medical undergraduates (MBBS [Bachelor's in Medicine, and Bachelor's in Surgery], Bsc nursing [Bachelor of Science in Nursing] and PCL nursing [Proficiency Certificate Level in Nursing]) of Lumbini Medical College and Teaching Hospital (LMCTH) studying in the 1^st^ year to those who were doing the Internship in the same hospital were taken as the target population. Students were informed about the study before handing the questionnaire, and their willingness to participate in the survey was considered as the consent given for participation. Convenient sampling was used to enroll participants. Sample size was calculated using the formula,

$$\begin{array}{ll} \text{n} & \text{= }{\text{Z}}^{\text{2}}\times \text{p}\times \text{(1}-\text{p)}/{\text{e}}^{\text{2}}\\ & \text{= }{\left(1.96\right)}^{\text{2}}\times (0.5)\times (1-0.5)/{(\text{0.05)}}^{\text{2}}\\ & \text{= }384.16 \end{array}$$

Where,

n = required sample size
Z = 1.96 at 95% Confidence Interval
p = population proportion, 50%
e = margin of error, 5%

Considering a non-respondent rate of 20%, a final sample size of 461 was taken for the study.

The participants who had heard about the guidelines were defined as those who had idea about the steps of the WHO hand hygiene guidelines, and were aware of the content of guideline, including the How, When and Why part of WHO brochure.^11^ Similarly, the participants who were habituated with the guidelines were identified as those respondents who followed the guidelines during each hand wash, while washing hands with soap and water.

The questionnaires were administered through an online Google survey form, which was made available through email and social media (Facebook, Messenger, What'sapp and Viber). The subjects were categorized into different groups based on their age group, the field of study, and also their year of study. All the subjects were informed about the confidentiality of their personal information, as well as even of their right to drop-out of the study, any time within the study period. Nevertheless, the following bias could inevitably occur. As, this was online survey, many bias still persisted; however, some bias, like social desirability bias was eliminated by keeping the information of respondents anonymous.

As the survey was done through online questionnaires, only those forms with complete data were accepted. The responses to all the questions were processed and analyzed by using simple descriptive statistics (in Microsoft excel). The results thus obtained were expressed in terms of percentage and presented using a pie chart.

## RESULTS

In our study, a total of 462 respondents participated, and we achieved a response rate of 98.2%. Among which, the majority of respondents belonged to 20-25 age groups, 275 (59.5%), and the majority were pursuing an MBBS degree, 360 (77.9%).

Of 462 respondents in the survey, a total of 261 (56.5%) respondents had recently (from Jan 2019- May 2020) been acquainted with the WHO hand-washing guidelines; moreover, 172 (37.2%) had learned the guidelines through awareness program in TV or social media, while the remaining 89 (19.2%) had acquired the knowledge about the guidelines through some other sources (through school teaching, course-book or from seniors), which showed that a notable number of participants seem to have acquired knowledge from awareness programs in recent years.

**Figure 1:**
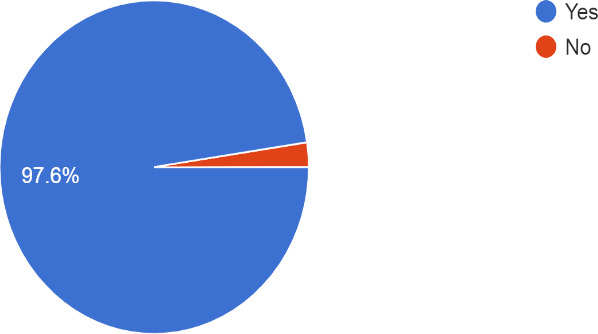
The medical undergraduates who have heard about WHO hand hygiene guidelines.

The participants who had heard about WHO hand-washing guidelines were 451 (97.6%); however, 430 (93.11%) participants knew about the WHO hand-washing guidelines before the COVID-19 outbreak (Figure 1).

**Figure 2:**
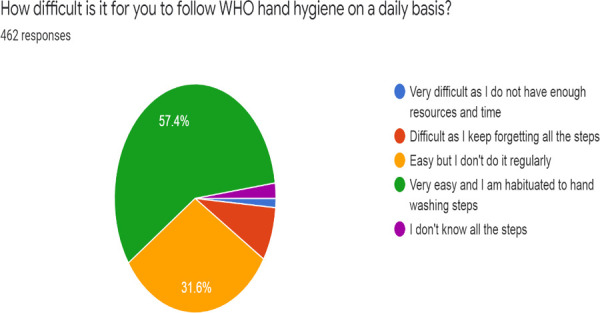
Responses to the difficulty level of following WHO hand washing guidelines.

Maximum respondents, 265 (57.4%), found the WHO hand-washing guidelines very easy to follow and were habituated to follow those guidelines, 146 (31.6%) respondents thought that these guidelines were easy to follow but they didn't follow it during every hand wash, 35 (7.6%) respondents found it difficult to follow those guidelines as they kept forgetting all the steps, while 10 (2.2%) respondents didn't know the guidelines at all and 6 (1.3%) knew the guidelines but found it very difficult to follow due to lack of resources and time (Figure 2).

**Figure 3:**
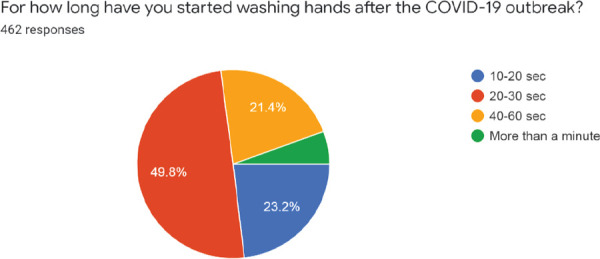
Time taken to wash hands with soap and water during a single hand wash.

Moreover 441 (95.5%) had access to clean water, and soap for hand washing or hand sanitizer for hand-rub, while 21 (4.5%) had no access to hand-washing resources which could be a reason why WHO hand-washing guidelines were not followed by all the respondents. Nonetheless, even if 265 (57.4%) respondents followed the WHO guidelines and were habituated, only 99 (21.4%) respondents washed their hands for 40-60 seconds, which is the standard length of time for washing hands (with soap and water) according to the guidelines; maximum respondents, 230 (49.8%), washed their hands only for 20-30 seconds, 107 (23.2%) washed their hands for 10-20 seconds and 26 (5.6%) washed their hands for more than a minute (Figure 3).

Further, 223 (48.2%) of the respondents gave their opinion on how hand-washing could be encouraged among the general public. Many, 216 (46.7%), responded that awareness program both in small scale and large scale should be launched in different platforms, including in social media platforms as well; moreover, they believed that awareness was still lacking in our community, and even if people knew about the guidelines they did not follow it while washing hands because they were not conscious about the severe side effects of unclean hands. Five (1.08%) respondents suggested that touting pamphlets and signs of WHO hand-washing guidelines in prominent locations throughout schools all over the nation could encourage hand washing. Two (0.43%) respondents thought that teaching these guidelines as a part of the school curriculum from a very young age could bring a good result in hand-washing practice.

## DISCUSSION

One of the best preventive measures for protecting ourselves from the risk of getting infected from the corona virus is washing hands properly and efficiently.^12^ Moreover, medical personals that come in direct contact with the patients have a high chance of spreading the virus and nosocomial infections; therefore, it is of utmost necessity that they know proper hand washing technique and that they wash hands regularly following standard hand washing guidelines. But, there are some pivotal shortcomings; major being the health professionals themselves not following the WHO hand washing guidelines or not having proper knowledge about the guideline. Therefore, in order to stop or slow down the rapid spread of virus, it seems very crucial for medical students to practice proper hand washing, so they can be habituated during their learning phase and can practice these hand washing techniques while serving as a medical professionals in future as well.

In our study, 97.6% of medical undergraduates had heard about the WHO hand-washing guideline, which is very high compared to a similar study done by Tze- Wai Wong, et al. in Hongkong,^13^ during SARS epidemic, in which only 51.5% knew about the WHO hand-washing guidelines. The increase in hand hygiene practice could be accounted for multiple factors, one of them being awareness among people through various media. Similar findings were seen in the study during SARS epidemic done in Hongkong,^13^ in which 35.2% of the students washed their hands before and 72.3% washed their hands after the physical examination of the patients (before knowledge of SARS) and after the spread, 60.3% (25.1% increase) washed hands before and 100% (27.5% increase) after the physical examination of the patient. Additionally, a study^14^ done post-Ebola also showed 10% of the respondents had imbibed hand-washing habits and the use of hand sanitizers during the Ebola virus disease outbreak.

Different types of awareness programs have been launched both by the governmental and non-governmental organization to make people aware about the precautions to be followed to avoid getting infected, these precautions included washing hands according to the WHO guidelines, wearing a mask, and social distancing. Even in schools, colleges, hospitals, and other institutions, these precautions were taught to make as many people aware as possible. These awareness programs seem to have been effective as among those undergraduates who had recently learned the guidelines 37.2% acquired the knowledge from awareness programs (through TV, radio, or social media); additionally, the study of E. Seimetz, et al.^15^ also showed an increase from 3.98 mean to 4.09 mean in the intention of washing hands after awareness-raising campaign. Furthermore, a study conducted by S Sulaiha S A, et al,^16^ also found out that despite a high level of knowledge, and awareness in medical students there was a lack of proper and effective hand washing practice.

The prevalence of medical undergraduates who followed WHO guidelines during each hand wash was found to be 57.4% in our study; however, in a research done in health professionals of Northeast Ethiopia,^17^ it was found that even if 65.9% were knowledgeable about the hand-washing only 43.0% followed it in a regular basis. There are many factors due to which people may not opt to follow the WHO hand hygiene guidelines even if they know it and one of the major factors could be a lack of proper hand-washing resources (clean water, soap, and hand sanitizer for hand-rub).

Although, only 27% of the population has access to safely managed drinking water and only 62% of people have access to at least basic sanitation services in the nation (Nepal),^18^ 95.5% of the undergraduates in our study responded that they had enough resources for hand hygiene (which includes water, soap or hand sanitizer for hand rub). Highlighting the fact that the priority of hand-washing among people has increased due to which they might have gathered required resources for proper hand hygiene, despite the challenges.

According to the standard WHO hand-washing guidelines, the duration of the entire hand washing procedure is 40-60 seconds while that of the entire hand rub procedure (with hand sanitizer) is 20-30 seconds. In our study, the majority of the respondents (49.8%) washed their hands only for 20-30 seconds. Even though 57.6% of medical undergraduates claim to follow the guidelines, they do not seem to follow it correctly and only 21.4% seem to follow the guidelines sincerely (i.e. wash their hands for 40-60 seconds). Similar results were seen in the study done by Grace M. Mbouthieu Teumta, et al.^19^ in which 56.7% of the respondents knew about hand hygiene, but more than half (75.2%) did not follow proper hand-washing techniques. Even in a study done in medical residents in Iran,^20^ the residents had moderate knowledge but overall poor attitudes and practices. The reason behind such an error could be a lack of proper awareness. Even if awareness programs are being held, it might not have been effective enough to leave a correct and deep impression to change the behavior of people.

In our study, 57.4% of the undergraduates found the WHO guidelines very easy to follow and they followed it in each hand wash; however, 7.6% of the respondents found it quite difficult as they have difficulty remembering all the steps of the WHO hand-washing guideline. A study was done by Tschudin-Sutter S, et al.^21^ to find out whether simplifying the WHO hand-washing steps could improve the tendency of proper hand-washing among people. The study found out that compliance with technique and indications of the 3-step hand-washing technique was 51.7% and 75.9%, respectively, as compared to 12.7% and 65.0% with the 6-step hand washing technique. Therefore, WHO hand-washing guidelines could be very beneficial if followed regularly, but as it is lengthy to follow, it might not be practical for all people to follow all the steps in each hand-wash. So, it was seen that replacing the long steps with short, easy, but effective steps could make the guidelines practically applicable to be followed during every hand wash.

This study is a cross-sectional study which was done through an online survey during the COVID-19 lockdown period of the country. There are some inevitable limitations in our study because it was not possible to go to the field for data collection; similarly, the self-reported data by the respondents were not very accurate in our study (for example- the time spent by the respondents during washing of hands was just approximated and the exact time calculation during each hand wash was not done). Furthermore, as this was an online survey, the questions that the respondents did not understand could not be explained to them, due to which few answers given by the respondents might have been unreliable. Moreover, the study gives a brief outlook of the handwashing tendency in medical undergraduates of a specific medical college in Nepal and cannot be used to generalize among the public. The participants were informed about the confidentiality and completely voluntary status before getting involved in the survey.

## CONCLUSIONS

A majority of medical undergraduates are acquainted with hand-washing according to the WHO guidelines, and the tendency of following all the steps has increased since the COVID-19 outbreak. However, some medical undergraduates seem to prefer simple hand-washing over following the WHO guidelines, mentioning that the guidelines are quite long to be followed in every hand wash. Similarly, proper hand hygiene awareness still seems to be lacking, as some well-educated medical undergraduates are still not motivated enough to follow appropriate hand hygiene techniques. Therefore, different organizations need to put on more effort to organize effective awareness programs, and hand hygiene should be included in the medical curriculum as medical undergraduates are the future health professionals, with the responsibility to take care of the publics' health in general.
